# Responses of *Panax notoginseng* (Burk.) F.H. Chen to cadmium stress: hormetic effects on growth, antioxidant systems, and rhizosphere microbial dynamics

**DOI:** 10.3389/fmicb.2026.1741415

**Published:** 2026-02-18

**Authors:** Mulan Wang, Yuewen Huo, Yarui Zhao, Xuanxiang Du, Byung-Wook Yun, Jingheng Wu, Xin Ying, Fugang Wei, Yanlin Wang, Rui Lu, Jiaqi Chen, Xin Wang, Qinsong Yang, Li Liu

**Affiliations:** 1 Key Laboratory of Natural Products Synthetic Biology of Ethnic Medicinal Endophytes, State Ethnic Affairs Commission, Yunnan Minzu University, Kunming, Yunnan, China; 2 Key Laboratory of Chemistry in Ethnic Medicinal Resources, State Ethnic Affairs Commission and Ministry of Education, Yunnan Minzu University, Kunming, Yunnan, China; 3 Department of Applied Biosciences, College of Agriculture and Life Sciences, Kyungpook National University, Daegu, Republic of Korea; 4 Miaoxiang Notoginseng Company with Limited Liability, Yunnan, Wenshan, China; 5 Yunnan Minzu University, Kunming, Yunnan, China; 6 Germplasm Bank of Wild Species and Yunnan Key Laboratory of Crop Wild Relatives Omics, Kunming Institute of Botany, Chinese Academy of Sciences, Kunming, Yunnan, China; 7 Institute of Medicinal Plants, Yunnan Academy of Agricultural Sciences, Yunnan, China

**Keywords:** cadmium stress, co-occurrence network, functional redundancy, hormesis, *Panax notoginseng*, rhizosphere microbiome

## Abstract

**Background:**

Cadmium (Cd) contamination poses a major threat to *Panax notoginseng* (Burk.) F.H. Chen cultivation, yet the dose-dependent thresholds separating adaptive responses from toxicity remain poorly understood, particularly at the level of rhizosphere microbial processes.

**Methods:**

A 75-day pot experiment was conducted using eight soil Cd concentrations (0–100 mg kg^–1^). Plant growth traits and antioxidant enzyme activities (SOD, POD, CAT) were measured. Rhizosphere microbial communities were characterized in terms of alpha and beta diversity, co-occurrence network structure, and predicted functional potential using PICRUSt2 and FAPROTAX.

**Results:**

Moderate Cd exposure (∼30 mg kg^–1^) enhanced plant growth and antioxidant enzyme activities, whereas high Cd ( ≥ 80 mg kg^–1^) caused physiological inhibition, consistent with a hormetic response. Microbial alpha diversity also peaked under moderate Cd but declined sharply at high Cd levels. Beta diversity differentiation was primarily driven by shifts in relative abundance rather than taxonomic turnover. Severe Cd stress reduced co-occurrence network connectivity and increased negative correlations, although several persistent core taxa (e.g., *Granulicella*) were maintained. Functional predictions indicated substantial functional redundancy, with key nutrient-cycling pathways largely retained despite pronounced network simplification.

## 1 Introduction

Cadmium (Cd) is a highly toxic heavy metal pollutant of global concern due to its mobility, bioavailability, and persistence in soils (Jiang et al., 2020; Singh and Chandel, 2022). The International Agency for Research on Cancer (IARC) classifies Cd as a Group 1 carcinogen, underscoring its severe risks to human health (Yu et al., 2021). In soils, Cd exists mainly in ionic forms easily absorbed by roots, resulting in bioaccumulation in crops. This is particularly problematic for traditional Chinese medicine (TCM) plants, where even trace residues compromise safety and therapeutic efficacy. Surveys have revealed that Cd contamination in TCM products exceeds permissible limits in 8–39% of samples across China (Shi et al., 2021; Yang et al., 2024; Mustafa and Komatsu, 2016), highlighting a pressing food safety issue.

China faces especially severe Cd contamination in agricultural soils, notably in provinces such as Yunnan, Hunan, and Guangxi. According to the national soil environmental quality standard (GB 15618–2018), the screening value and intervention threshold for Cd are set at 0.4 mg kg^–1^ and 0.6 mg kg^–1^, respectively, for soils with pH ranging from 5.5 to 7.5 (GB 2760–2024) National Health Commission of the People’s Republic of China (2024). However, extensive field investigations in mining- and industry-impacted regions consistently report Cd concentrations far exceeding these thresholds, commonly reaching 20–50 mg kg^–1^ in farmland soils and exceeding 100 mg kg^–1^ in contamination hotspots (Chen et al., 2015; Zhao et al., 2010). Such elevated Cd levels pose severe threats not only to crop productivity but also to soil ecological stability and long-term agroecosystem sustainability.

Cd stress adversely affects plant physiology by disrupting antioxidant defense systems, inhibiting growth, and reducing biomass accumulation (Shi et al., 2016; Song et al., 2023; Yang et al., 2021; Zhao et al., 2021). Typical symptoms include chlorosis, growth retardation, and yield loss. Beyond its direct phytotoxic effects, Cd contamination also profoundly alters rhizosphere soil functioning. Microbial communities and soil enzymes, which play central roles in nutrient cycling and energy metabolism, are particularly sensitive to heavy metal stress (Bell et al., 2014; Caldwell, 2005; Shi et al., 2022; Song et al., 2021; Steinauer et al., 2015). Key enzymes such as urease, catalase, and acid phosphatase are often inhibited under elevated Cd levels, leading to reduced soil fertility and impaired nutrient availability (Aponte et al., 2020; Yu et al., 2006). Moreover, Cd exposure frequently restructures rhizosphere microbial communities, characterized by declines in metal-sensitive taxa and enrichment of metal-tolerant populations, thereby reshaping soil ecological processes (Chodak et al., 2013; Deng et al., 2015; Ding et al., 2017).

Importantly, plant growth–promoting rhizobacteria (PGPR) can alleviate Cd stress by reducing Cd bioavailability and enhancing plant tolerance. Proposed mechanisms include Cd immobilization/chelation in the rhizosphere, modulation of rhizosphere chemistry, and stimulation of plant growth and stress defenses via phytohormones, ACC deaminase, siderophores, and antioxidant-related support (Rajkumar et al., 2009). Representative genera such as *Pseudomonas* and *Bacillus* have been reported to contribute to Cd mitigation and improved plant performance in contaminated soils (Suksabye et al., 2016). Recent evidence further suggests that PGPR may enhance Cd phytoremediation by reshaping rhizosphere microbial partnerships and functional processes at the community level (Chi et al., 2025; Ercole et al., 2025).

Hormesis, a biphasic dose–response phenomenon, has attracted increasing attention in plant ecotoxicology. Low Cd exposure may stimulate beneficial responses such as enhanced photosynthesis and stress tolerance, whereas higher concentrations lead to growth inhibition or even plant death. Understanding such threshold-dependent responses is therefore critical for optimizing stress management and ensuring sustainable cultivation. Recent advances indicate that Cd stress responses are not solely plant-intrinsic but are strongly modulated by rhizosphere microbial processes. Integrated multi-omics approaches have shown that Cd exposure can reshape root exudation profiles and selectively recruit beneficial microorganisms, thereby regulating rhizosphere pH, metal bioavailability, and microbial metabolic networks, ultimately contributing to plant detoxification and stress buffering (Jing et al., 2025). In parallel, studies on soil microbiomes have emphasized that functional redundancy, in which multiple taxa share overlapping functional capabilities, can stabilize ecosystem functioning under disturbance, allowing key biogeochemical processes to persist even when community composition and interaction structure are reorganized (Jurburg and Falcao Salles, 2015). In the context of this study, hormesis is primarily considered a plant-level physiological response, whereas changes in soil enzyme activities and rhizosphere microbial communities are examined as coordinated, threshold-dependent processes potentially coupled to plant stress responses rather than as independent manifestations of hormesis. However, despite the economic and medicinal importance of *Panax notoginseng* (Burk.) F.H. Chen, its threshold-dependent responses to Cd stress, which are particularly mediated by rhizosphere microbial processes and functional redundancy, remain poorly understood.

*P. notoginseng* is a high-value medicinal plant that is particularly sensitive to soil environmental quality. As a root-harvested medicinal crop, *P. notoginseng* is directly exposed to soil-borne contaminants, and even low levels of Cd accumulation can compromise medicinal safety and marketability due to the stringent regulatory standards applied to traditional Chinese medicine. Previous studies have reported a typical hormetic response of *P. notoginseng* to Cd stress, with stimulation at low Cd levels and inhibition at higher Cd concentrations, accompanied by dose-dependent changes in the rhizosphere microbial community (Liu et al., 2025), underscoring its suitability as a model species for investigating threshold-dependent Cd stress responses and rhizosphere microbial processes in medicinal crops.

However, it remains unclear whether Cd-induced hormetic responses in *P. notoginseng* are confined to plant physiological traits or are accompanied by coordinated changes in rhizosphere enzyme activities, microbial community structure, and functional interactions. In particular, the extent to which rhizosphere microbial processes contribute to buffering Cd stress across hormetic and toxic thresholds remains poorly understood. We hypothesized that moderate Cd exposure would induce coupled plant–microbe buffering responses, whereas excessive Cd stress would disrupt microbial network connectivity and functional stability. To test these hypotheses, we systematically investigated the effects of a gradient of Cd concentrations on plant growth performance, antioxidant enzyme activities, and rhizosphere microbial communities. By integrating agronomic measurements, physiological assessments, microbial diversity analyses, and co-occurrence network and functional predictions, we aimed to elucidate how plant–microbe interactions respond to Cd stress across hormetic and toxic thresholds, thereby providing a theoretical basis for the sustainable cultivation of *P. notoginseng* in Cd-contaminated soils.

## 2 Materials and methods

### 2.1 Overview of the test site

This experiment was conducted at the *P. notoginseng* Science and Technology Park in Miao Township, Yanshan County, Yunnan Province, China (104.39° E, 24.71° N). The region features a subtropical plateau monsoon climate with an average annual temperature of 16.1°C and annual precipitation of 1008 mm.

### 2.2 Experimental design

Uniformly cultivated 7-month-old *P. notoginseng* seedlings were selected and transplanted into pots containing 10 kg of cultivation soil, with a total of 45 seedlings per pot. Eight Cd treatments 0 mg kg^–1^(CK), 10 mg kg^–1^ (N1P), 20 mg kg^–1^ (N2P), 30 mg kg^–1^ (N3P), 40 mg kg^–1^ (N4P), 60 mg kg^–1^ (N5P), 80 mg kg^–1^ (N6P), 100 mg kg^–1^ (N7P) were applied, each with three replicates. Cd was applied as CdCl_2_⋅2.5H_2_O solutions uniformly mixed into the soil. During the experimental period, plants were maintained under natural field conditions with supplemental shade netting (70% light transmission) and were watered every 2–3 days to maintain soil moisture at 60–70% of field capacity, with no additional fertilizers applied. The Cd concentration gradient was designed to cover environmentally relevant contamination levels as well as low- and high-dose ranges, enabling assessment of both hormetic and toxic responses.

### 2.3 Sample collection, processing, and analytical methods

After 75 days of treatment (from August 15, 2022, to October 31, 2022), *P. notoginseng* plants were harvested as whole plants, including both aerial parts and roots. The whole plants were thoroughly washed with deionized water. For each treatment, 15 plants were randomly selected for enzyme activity assays, while the remaining plants were used for agronomic trait measurements. After agronomic trait measurements, the stems, leaves, and roots were separated and initially heat-treated at 105°C for 30 min, followed by drying at 65°C for 6 h until reaching a constant weight. The dried samples were then ground into fine powder for Cd content determination.

Fresh soil samples were collected and immediately placed in sterile centrifuge tubes, transported to the laboratory in an icebox, and stored at –80°C for 16S rRNA sequencing. The remaining soil samples were air-dried indoors and sieved through 40-mesh and 100-mesh screens for enzyme activity and cadmium content analysis, respectively.

Agronomic traits were measured using a ruler with a precision of 0.1 cm. Plant height was determined as the length from the cutting site to the petiole base. Leaf length (central leaflet length), leaf width (central leaflet width), and leaf area (calculated as leaf length × leaf width × 0.6134) were recorded. Root fresh weight was measured using an analytical balance with a precision of 0.01 g.

Enzyme activity assays were conducted using commercial assay kits (Beijing Solarbio Science & Technology Co., Ltd., Beijing, China) following the manufacturer’s protocols. Antioxidant enzyme activities in plant tissues, including superoxide dismutase (SOD), peroxidase (POD), and catalase (CAT), were determined using the nitroblue tetrazolium (NBT) photoreduction method, the guaiacol method, and the ammonium molybdate method, respectively. Soil enzyme activities, including acid phosphatase (S-ACP), urease (S-UE), and catalase (S-CAT), were measured using the disodium phenyl phosphate method, the indophenol blue colorimetric method, and the hydrogen peroxide decomposition method, respectively. Enzyme activities were expressed on a fresh weight basis for plant tissues and a dry weight basis for soil samples, according to the instructions provided with each assay kit.

The Cd concentrations in soil and *P. notoginseng* samples were analyzed using graphite furnace atomic absorption spectrometry (GFAAS) (Beijing Purkinje General Instrument Co., Ltd., Beijing, China). Survival rate was defined as the percentage of plants remaining alive at the end of the 75-day Cd treatment period relative to the initial number of plants per treatment. The bioaccumulation coefficient (BCF) was calculated as the ratio of Cd concentration in individual plant tissues to the corresponding Cd concentration in soil. The translocation coefficient (TC) was calculated as the ratio of Cd concentration in aboveground tissues to that in belowground tissues.

### 2.4 Rhizosphere soil sampling

To isolate rhizosphere soil, whole plants were gently uprooted, and loosely adhered soil was removed by carefully shaking the roots. Rhizosphere soil tightly attached to the roots (defined as soil within 1–2 mm of the root surface) was then collected by brushing the root surface with a sterile brush and shaking vigorously for 2 min in 10 mL of phosphate-buffered saline (PBS). The resulting soil suspension was centrifuged at 5,000 rpm for 10 min, and the pellet was used for downstream DNA extraction.

### 2.5 Soil DNA extraction

Total genomic DNA was extracted from 0.25 g of rhizosphere soil using the TIANGEN^®^ Soil DNA Kit (TIANGEN, Beijing, China), following the manufacturer’s protocol based on CTAB/SDS lysis chemistry. This kit has been widely used for microbial DNA extraction from soil samples with high efficiency and purity (Zhou et al., 1996). DNA concentration was measured using a Qubit^®^ 2.0 Fluorometer (Thermo Fisher Scientific, United States), and quality was verified by 1% agarose gel electrophoresis. DNA was diluted to 1 ng μL^–1^ with sterile water for downstream amplification.

### 2.6 16 S rRNA Gene amplification, library preparation, and sequencing

The V3–V4 hypervariable region of the bacterial 16S rRNA gene was amplified using the primer pair 341F (5’-CCTAYGGGRBGCASCAG-3’) and 806R (5’-GGACTACNNGGGTATCTAAT-3’), which target a broad range of bacterial taxa. PCR reactions were conducted in triplicate using Phusion^®^ High-Fidelity PCR Master Mix with GC Buffer (New England Biolabs, United States) (Avershina et al., 2013). PCR products were purified using AMPure XP beads (Beckman Coulter, United States), quantified with a Qubit dsDNA HS Assay Kit (Thermo Fisher Scientific, United States), and pooled in equimolar concentrations. Sequencing libraries were prepared using the TruSeq^®^ DNA PCR-Free Sample Preparation Kit (Illumina, United States) and sequenced on the Illumina NovaSeq 6000 platform using a paired-end 2 × 250 bp configuration.

### 2.7 Bioinformatic and statistical analysis

Raw reads were quality-filtered, demultiplexed, and processed using QIIME2 (v2023.2). Operational taxonomic units (OTUs) were clustered at 97% sequence similarity using the Uparse algorithm (USEARCH v7), and taxonomy was assigned using the Mothur method against the SILVA 138.1 reference database with a confidence threshold of 0.8. Rarefaction curves were generated to assess sequencing depth. Alpha diversity indices (Chao1, Shannon) and beta diversity metrics (Bray-Curtis, weighted UniFrac) were calculated. Principal coordinate analysis (PCoA) and hierarchical clustering (UPGMA) were used to visualize differences in community composition among treatments. Differential microbial biomarkers were identified and microbial co-occurrence networks were constructed following established analytical frameworks described by Banerjee et al. (2018). Differences in beta diversity among treatments were assessed using PERMANOVA (adonis) based on UniFrac distance matrices (*p* < 0.05).

### 2.8 Biomarker identification and network analysis

Differential microbial taxa among treatment groups were identified using LEfSe (Linear Discriminant Analysis Effect Size), implemented on the Galaxy web platform.^1^ The analysis followed the standard pipeline: first, the Kruskal–Wallis rank-sum test was used to detect features with significant differential abundance (α = 0.05); this was followed by unpaired Wilcoxon rank-sum tests for pairwise comparisons. Finally, Linear Discriminant Analysis (LDA) was applied to estimate the effect size of each differentially abundant feature, using an LDA score threshold of 4.0. LEfSe was applied to genus-level relative abundance data, unless otherwise specified. Results were visualized using bar plots and cladograms to highlight the most discriminative microbial biomarkers across groups.

### 2.9 Microbial co-occurrence network analysis

Co-occurrence network analysis was performed using SparCC to assess microbial interactions under cadmium stress. Networks were constructed separately for control (N0P: 0 mg kg^–1^ Cd) and high Cd stress (N7P: 100 mg kg^–1^ Cd) conditions to evaluate Cd-induced changes in bacterial community interactions. The N6P treatment was selected as it showed the strongest Cd-induced community shifts. Bacterial genus-level OTU data were filtered to include genera with > 0.1% relative abundance in ≥ 50% of samples per treatment. SparCC correlations were computed with 100 bootstrap replicates, and only statistically significant correlations (| ρ| > 0.8, *p* < 0.05) were retained. Network topology parameters (node degree, betweenness centrality, modularity) were calculated using igraph in R. Networks were visualized in Gephi v0.9.2 with Force Atlas 2 layout. Nodes represent genera (colored by phylum, sized by degree centrality), while edges indicate positive (red) or negative (blue) correlations with thickness reflecting correlation strength. Network topology differences between treatments were quantitatively compared to assess heavy metal impacts on microbial community structure.

### 2.10 Statistical analysis of data

Data were analyzed using SPSS 22.0, with one-way ANOVA applied to assess significant differences (*p* < 0.05). Results were represented as means with corresponding groupings indicated by lowercase letters.

## 3 Results

### 3.1 Moderate Cd levels enhance *P. notoginseng* growth and antioxidant defense, whereas high Cd induces phytotoxicity

**FIGURE 1:**
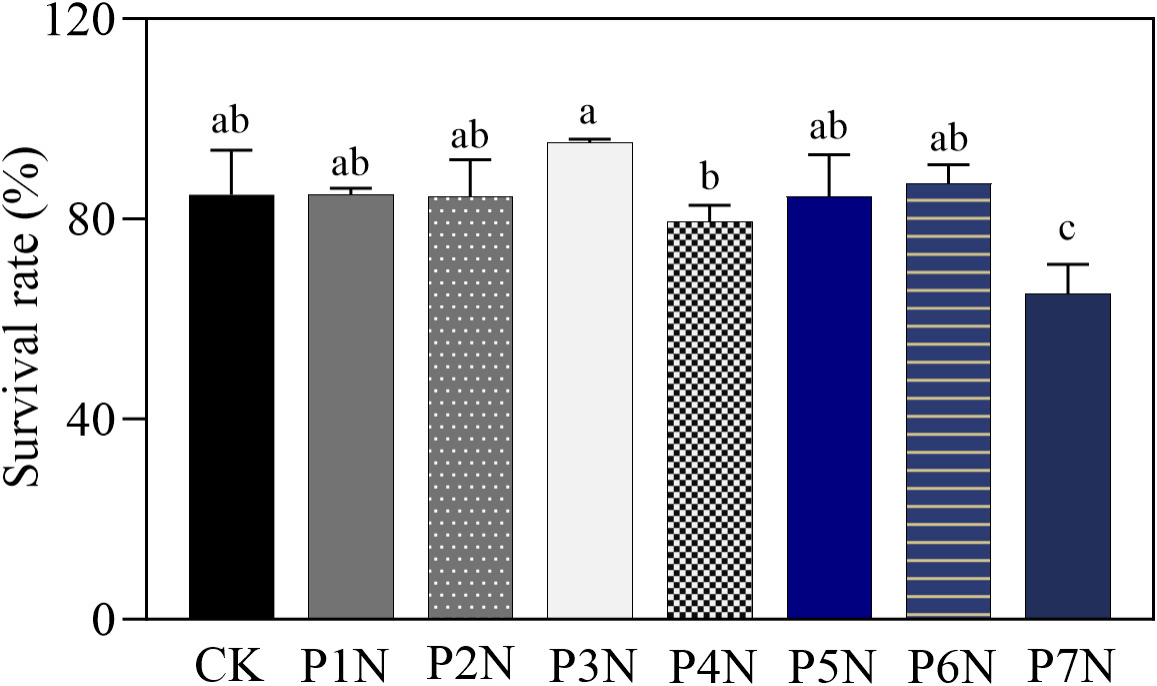
Survival rate of *P. notoginseng* after 75 days of Cd treatment. Survival rate represents the percentage of plants remaining alive at the end of the treatment period. Different letters above bars indicate statistically significant differences among treatments based on ANOVA with *post-hoc* multiple comparisons (*p* < 0.05). Error bars represent the standard deviations (SD) of the mean (*n* = 3).

The growth performance of *P. notoginseng* showed a clear hormetic response to Cd exposure. Plant height, leaf length, leaf width, leaf area, and root fresh weight increased under moderate Cd levels (N2P–N3P; ∼30 mg kg^–1^ Cd), but declined markedly under high Cd (N7P) (Table 1 and Supplementary Figure 1). Survival rate followed a similar non-linear trend: moderate Cd (N3P) improved survival to 95.34 ± 0.65%, whereas excessive Cd (N7P) reduced it to 65.07 ± 5.91% (Figure 1). Survival rate, defined as the percentage of plants remaining alive after 75 days of Cd treatment, varied among Cd treatments, with the highest survival observed at the moderate Cd level (N3P) and a marked decline under excessive Cd stress (N7P) (Figure 1).

**TABLE 1:** Effects of Cd stress on growth indices of *P. notoginseng* (*n* = 60).

Treatments	Plant height (cm)	Leaf length (cm)	Leaf width (cm)	Leaf area (cm^2^)	Root weight (g)
CK	9.87 ± 0.81 ab	4.84 ± 0.37 ab	2.45 ± 0.32 bc	7.18 ± 1.16 bc	1.24 ± 0.30 bc
N1P	9.54 ± 0.88 cd	4.80 ± 0.62 ab	2.43 ± 0.36 bcd	7.00 ± 1.32 bcd	1.26 ± 0.35 bc
N2P	9.65 ± 0.91 bc	4.83 ± 0.59 ab	2.48 ± 0.34 ab	7.23 ± 1.22 b	1.41 ± 0.31 a
N3P	9.99 ± 1.09 a	4.87 ± 0.58 a	2.51 ± 0.45 a	7.46 ± 1.17 a	1.34 ± 0.32 ab
N4P	9.65 ± 0.96 bc	4.82 ± 0.63 ab	2.47 ± 0.36 ab	7.21 ± 1.21 b	1.31 ± 0.33 ab
N5P	9.50 ± 1.01 cd	4.79 ± 0.68 ab	2.45 ± 0.36 bc	7.12 ± 1.17 bc	1.23 ± 0.29 bc
N6P	9.48 ± 1.00 cd	4.76 ± 0.67 bc	2.42 ± 0.38 cd	6.97 ± 1.32 cd	1.19 ± 0.36 bc
N7P	9.30 ± 1.09 d	4.69 ± 0.82 c	2.39 ± 0.34 d	6.88 ± 1.25 d	1.11 ± 0.28 c

Values are means ± SD. Different letters indicate a statistically significant difference (*p* < 0.05).

**FIGURE 2:**
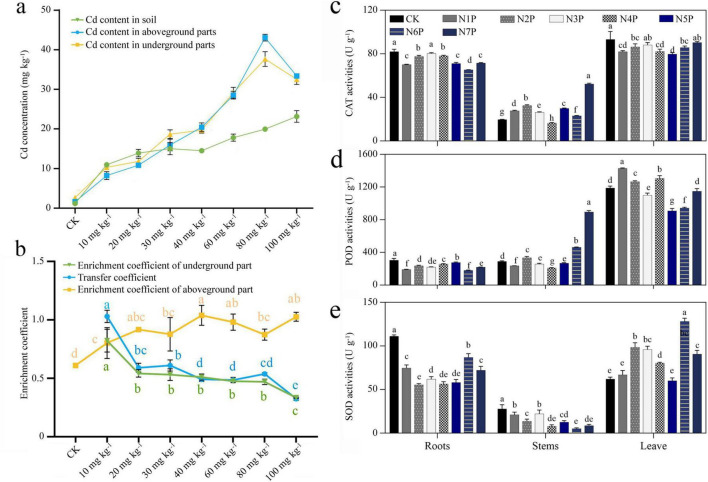
Cd accumulation and antioxidant enzyme activities in *P. notoginseng* under Cd stress. **(a)** Cd concentrations in soil, shoots, and roots. **(b)** BCF, TC, and enrichment coefficients of shoots and roots. **(c–e)** Antioxidant enzyme activities in CAT, POD, and SOD in different plant tissues. For panels **(b–e)**, different letters denote significant differences among treatments based on one-way ANOVA (*p* < 0.05). Error bars represent standard deviations (SD) (*n* = 3).

Cd accumulated primarily in roots, indicating a sequestration strategy to protect aerial tissues (Figure 2a). The BCF decreased with increasing Cd, while the TC peaked at N4P (1.04) before stabilizing (Figure 2b). Antioxidant enzyme activities (CAT, POD, and SOD) showed non-linear and tissue-specific responses to Cd stress, with marked induction at low Cd levels (N1P–N3P) and altered regulation under higher Cd exposure (N5P–N7P) (Figures 2c–e). Collectively, the results indicate that moderate Cd exposure elicits activation of defense-related physiological responses, while excessive Cd stress is associated with growth inhibition and pronounced changes in antioxidant enzyme activity patterns.

### 3.2 Moderate Cd is associated with enhanced soil enzyme activity and microbial diversity, whereas excessive Cd coincides with reduced rhizosphere function

**FIGURE 3:**
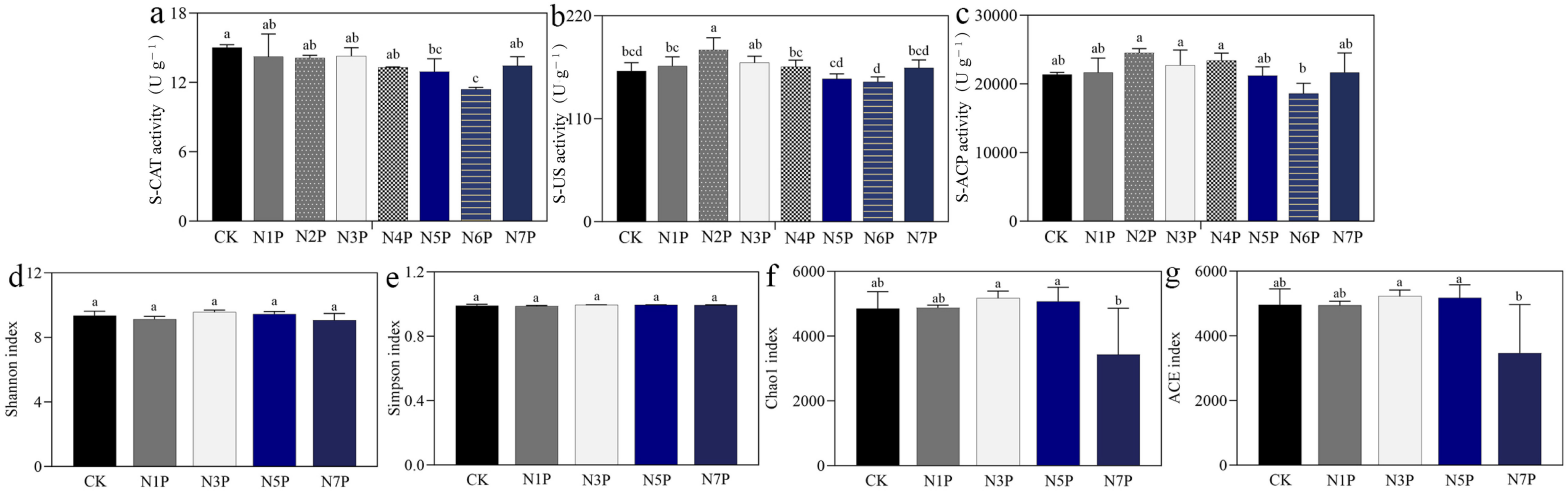
Effects of Cd stress on soil enzyme activities and microbial alpha diversity. **(a–c)** Soil enzyme activities: **(a)** S-CAT, **(b)** S-URE, **(c)** S-ACP. **(d–g)** Alpha diversity indices of rhizosphere microbes: **(d)** Shannon, **(e)** Simpson, **(f)** Chao1, **(g)** ACE. Different letters denote significant differences among treatments (ANOVA, *p* < 0.05). Error bars show SD (*n* = 3).

Soil enzymatic responses to Cd stress were non-linear and threshold-dependent (Figure 3). S-CAT activity declined steadily across treatments, whereas S-URE and S-ACP increased under moderate Cd (N3P–N5P) before decreasing under higher Cd (N7P). Such biphasic patterns suggest that low Cd may transiently enhance nutrient cycling through stimulated microbial activity, while high Cd suppresses key biochemical processes necessary for soil fertility.

In parallel, microbial alpha diversity indices (Shannon, Chao1, ACE) peaked under moderate Cd and declined sharply at N7P (Figures 3d–g), whereas the Simpson index remained relatively stable. Together, these data suggest that moderate Cd (30–60 mg kg^–1^) is associated with enhanced microbial and enzymatic activity but excessive exposure compromises rhizosphere stability and nutrient availability.

### 3.3 Cd stress restructures microbial diversity through abundance shifts rather than species turnover

**FIGURE 4:**
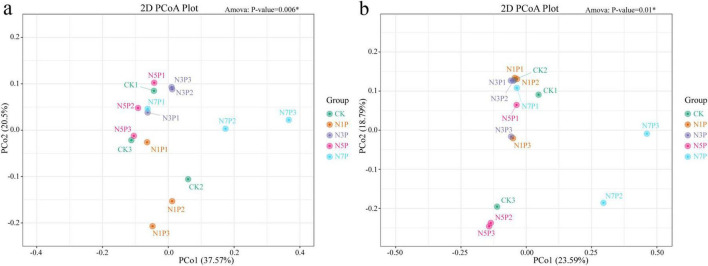
Beta-diversity analysis of microbial communities based on weighted **(a)** and unweighted **(b)** UniFrac distances using principal coordinates analysis (PCoA). Samples are colored by Cd treatment groups. The first two principal coordinates explain the indicated percentages of total community variance. PERMANOVA (adonis) *p*-values indicate significant treatment effects.

Beta-diversity analyses (weighted and unweighted UniFrac) demonstrated distinct community clustering across Cd gradients (Figure 4). Control (CK) samples were clearly separated from Cd-treated groups, and PERMANOVA (adonis) confirmed significant compositional differences among treatments. These findings indicate that These results suggest that Cd-induced community differentiation is largely associated with shifts in relative abundance rather than extensive turnover of major taxa.

Beta-diversity analyses (weighted and unweighted UniFrac) showed clear ordination shifts across the Cd gradient (Figure 4). PERMANOVA (adonis) further indicated moderate-to-large effect sizes of Cd treatment on community dissimilarity across pairwise contrasts (*R*^2^ = 0.215–0.510 for weighted UniFrac and 0.490–0.785 for unweighted UniFrac; Supplementary Table 1), suggesting that Cd exposure is associated with substantial community differentiation. Overall, the observed separation is consistent with Cd-related changes in community composition, largely reflected by shifts in the relative abundance and/or presence–absence patterns of dominant lineages rather than wholesale replacement of major phyla.

**FIGURE 5:**
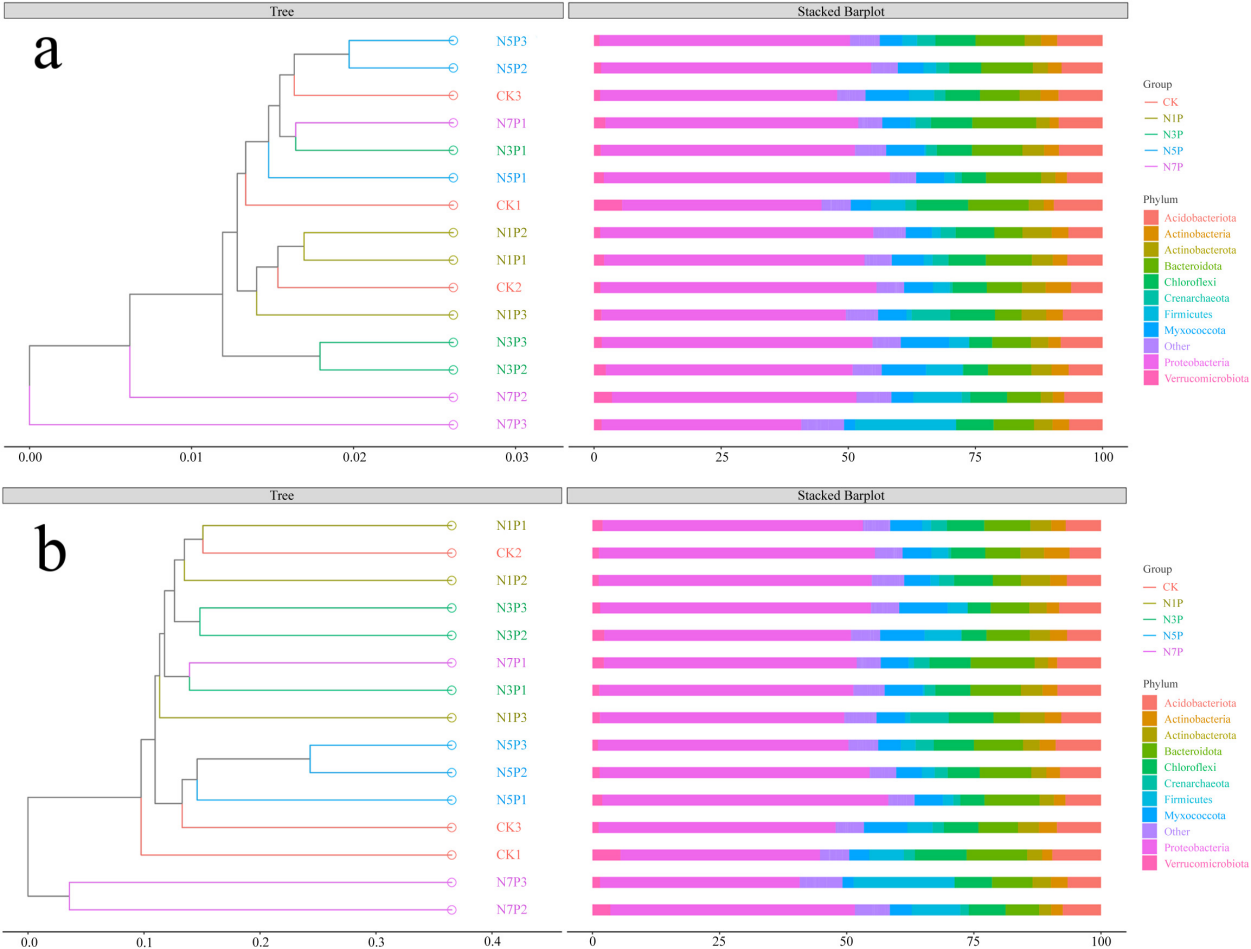
Phylogenetic clustering and taxonomic composition. **(a)** Weighted and **(b)** unweighted UniFrac dendrograms with phylum-level stacked bar plots. Branches are colored by treatment; scales indicate dissimilarity. Bars show relative abundance of major phyla.

Hierarchical and phylogenetic clustering (Supplementary Figure 2 and Figure 5) further supported this pattern by showing clear differentiation of microbial communities among Cd treatments, with high-Cd samples (N7P) generally tending to separate from the lower-Cd groups. Notably, some variability among biological replicates was observed within certain Cd treatment groups, suggesting heterogeneous community responses under Cd stress. Overall, these results indicate that Cd stress is associated with pronounced restructuring of community composition, which is largely reflected by shifts in the relative abundance of dominant lineages across treatments.

### 3.4 Changes in microbial network structure and functional redundancy under Cd stress

**FIGURE 6:**
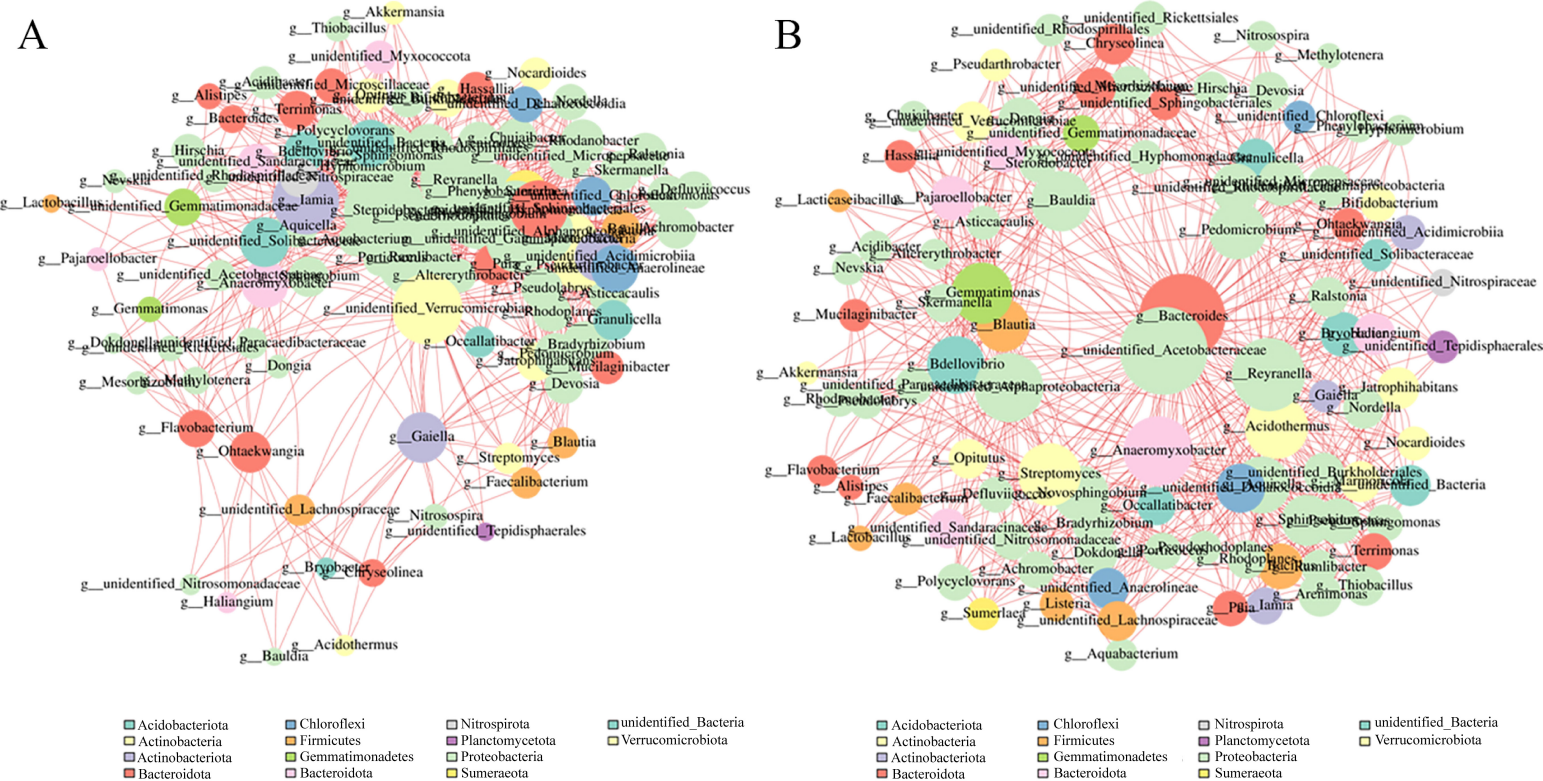
Co-expression networks of the rhizosphere bacterial communities under **(A)** CK and **(B)** N7P in *P. notoginseng*. Nodes represent bacterial genera (colored by phylum; size proportional to degree centrality). Edges indicate significant positive (green) or negative (red) correlations. To improve readability, only representative genera with relatively high degree centrality are labeled.

Cd stress altered the rhizosphere bacterial co-occurrence networks of *P. notoginseng* (Figure 6 and Supplementary Figure 6). Compared with the control, the high-Cd (N7P) network exhibited a higher proportion of negative correlations, with the proportion of negative correlations increasing from 36.7% in CK to 47.2% in N7P, as supported by quantitative network edge statistics (Supplementary Table 2). These quantitative differences indicate altered interaction patterns under Cd stress, consistent with a reorganization of the rhizosphere microbial network.

Along the Cd gradient, positive correlations declined while negative correlations increased, accompanied by higher modularity, indicating weakened connectivity and reduced buffering capacity under high Cd conditions. A global comparison (Supplementary Figure 7) further revealed that several persistent core taxa, particularly *Granulicella* and *Gaiella*, maintained high network connectivity across treatments. These taxa have been widely recognized as central members of soil microbial networks (e.g., Banerjee et al., 2018; Herren and McMahon, 2018), although their associations tended to become more antagonistic under Cd stress. Overall, these results suggest that Cd stress may transform rhizosphere microbial networks from more cooperative and redundant structures to increasingly specialized and competitive ones, with potential implications for rhizosphere ecological functioning under heavy metal contamination.

**FIGURE 7:**
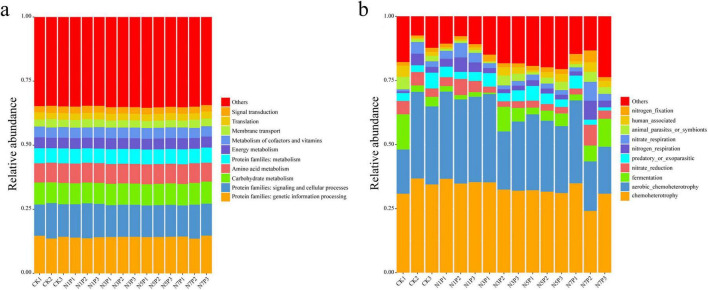
Predicted functional profiles under Cd stress. **(a)** PICRUSt2-predicted KEGG Level 2 functions. **(b)** FAPROTAX-annotated ecological functions. Values are relative abundances; statistical comparisons for selected functions are summarized in Supplementary Table 2 (mean ± SD, *n* = 3).

Functional predictions based on PICRUSt2 and FAPROTAX suggested a non-linear response of microbial functional profiles to Cd stress (Figure 7). At low Cd levels (N1P), the relative abundances of major functional categories remained comparable to the control, indicating a potential functional threshold. With increasing Cd exposure (N3P–N5P), subtle variations were observed in the relative abundances of functions associated with carbohydrate metabolism and xenobiotic-related processes, although these changes were not statistically significant, reflecting potential adaptive adjustments in microbial metabolism. Under high Cd stress (N7P), functions associated with environmental sensing and signal transduction tended to decline, whereas core metabolic functions, including energy, amino acid, protein, and xenobiotic metabolism, remained relatively stable across treatments.

Consistent with these trends, PICRUSt2-based statistical analysis showed that carbohydrate and energy metabolism did not differ significantly among treatments (one-way ANOVA, *p* > 0.05; Figure 7 and Supplementary Table 3), whereas signal transduction exhibited a significant decline with increasing Cd stress (*p* < 0.05), indicating that microbial environmental sensing and regulatory capacity is more sensitive to Cd exposure than core metabolic potential.

FAPROTAX ecological annotation further supported functional resilience, showing that chemoheterotrophy and aerobic chemoheterotrophy remained stable across the Cd gradient, ensuring continuous carbon turnover. Nitrogen cycling functions displayed adaptive plasticity: nitrogen fixation increased under high Cd stress, while nitrate and nitrogen respiration tended to be higher at low Cd levels, whereas fermentation and parasitic processes were reduced.

Together, these results suggest that functional redundancy and the maintenance of core metabolic functions may contribute to partial functional resilience in the *P. notoginseng* rhizosphere.

## 4 Discussion

### 4.1 Moderate Cd exposure induces hormetic growth stimulation, whereas high Cd triggers physiological inhibition in *P. notoginseng*

As the first level of Cd perception, plant physiological responses provide the primary driver shaping belowground processes. The present study showed that *P. notoginseng* exhibited a canonical hormetic response when exposed to Cd: moderate Cd (∼30 mg kg^–1^) promoted growth, improved leaf morphological traits, and stimulated antioxidant enzyme activities, whereas high Cd concentrations led to oxidative damage, biomass decline, and physiological inhibition. This pattern aligns with the broader consensus that hormesis is a fundamental response class in plants under environmental contaminants (Calabrese and Baldwin, 2003; Li R. et al., 2025; Saitanis and Agathokleous, 2019). Moderate Cd exposure may activate redox-linked signaling cascades (e.g., MAPK/ROS signaling loops) that prime antioxidant defense and transiently enhance photosynthetic efficiency and resource allocation (Carvalho et al., 2020). However, once Cd input exceeds detoxification capacity, excessive ROS accumulation overwhelms antioxidant buffering, leading to membrane peroxidation, impaired metabolic processes, and growth inhibition, as previously observed in other medicinal crops (Wang et al., 2023; Chen et al., 2024).

### 4.2 Root-based Cd sequestration buffers aerial toxicity but collapses beyond antioxidant defense thresholds

The strong root-biased Cd accumulation observed here suggests that *P. notoginseng* employs a retention strategy in which the roots function as the primary sequestration compartment, thereby restricting the translocation of Cd into aerial tissues, a trait of considerable economic importance given the medicinal use of its underground organs. Similar spatial partitioning of heavy metals between roots and shoots has been reported in *Arabidopsis* and other Cd-tolerant species (Lux et al., 2011; Ghori et al., 2019; Jia et al., 2020). At moderate Cd (∼30 mg kg^–1^), SOD, POD and CAT activities were markedly elevated, demonstrating that an effective stress-induced activation of antioxidant defense enzymes operates in *P. notoginseng.* Under high Cd however, these enzymes declined, consistent with enzymatic inactivation, protein carbonylation, and redox-homeostasis collapse (Lu et al., 2023; Mahmood et al., 2025). This exhaustion phase appears to coincide with a physiological transition where Cd enters beyond-threshold bioavailability, disrupting metabolic efficiency even in the presence of a rhizosphere microbial buffering effect.

### 4.3 Plant hormetic responses are coupled with threshold-dependent shifts in rhizosphere enzyme activity and microbial community structure

Root-based Cd sequestration and antioxidant regulation not only determine plant tolerance thresholds but also modulate the chemical and biological conditions of the rhizosphere. Although hormesis is classically defined at the level of organismal physiology, the present results suggest that plant hormetic responses to moderate Cd stress may be accompanied by coordinated shifts in rhizosphere enzyme activities and microbial community structure. Importantly, these microbial and enzymatic responses are interpreted as coupled feedbacks to plant stress and altered root exudation, rather than as direct evidence of microbial hormesis *per se*.

The nonlinear responses of rhizosphere enzymes and microbial alpha diversity indicate that Cd modulates belowground processes via threshold-dependent biochemical and ecological feedbacks. Moderate Cd (∼30 mg kg^–1^) enhanced urease and acid phosphatase activities and increased Shannon and Chao1 diversity, implying a transient acceleration of nutrient cycling, likely mediated by shifts in root exudation and stress-induced recruitment of beneficial microorganisms (Li N. et al., 2025; He et al., 2024). Rhizosphere network studies have shown that the architecture of trophic interactions largely determines the capacity of bacterial communities to buffer perturbation and resist pathogen invasion (Wei et al., 2020). However, high Cd sharply reduced enzyme activity and microbial richness, indicating that once toxicity exceeds functional buffering capacity, facilitative interactions weaken and rhizosphere metabolic turnover becomes impaired. The observation that beta diversity separation occurred mainly through abundance redistribution rather than phylum-turnover further supports the view that rhizosphere microbiomes are compositionally resilient yet functionally plastic only within definable tolerance boundaries (Griffiths and Philippot, 2013).

### 4.4 Microbial network stability, functional redundancy, and ecological resilience implications

Co-occurrence network analyses further revealed that Cd stress shifted rhizosphere microbial networks from cooperative-redundant architecture to more modular and competitive configurations, accompanied by reduced ecological connectivity. The densified positive interactions observed in control soils, which are characterized by cooperative taxa such as *Granulicella* and *Gaiella*, suggest the presence of a stable and metabolically buffered core network. These taxa are widely recognized as dominant and functionally important members of soil microbial communities, with *Acidobacteria* (including *Granulicella*) actively participating in carbon turnover and energy metabolism and contributing to soil organic matter processing (Kalam et al., 2020; Giguere et al., 2021; Delgado-Baquerizo et al., 2018). In addition, *Actinobacteria*, including *Streptomyces*, are well known for their roles in secondary metabolite production and nutrient cycling in soils, thereby supporting ecosystem functioning and resilience under non-stressed conditions (van der Meij et al., 2017).

Under high Cd stress, negative correlations increased and stress-tolerant hubs became more prominent, while PGPR genera such as *Pseudomonas* and *Bacillus* were selectively enriched, highlighting their well-characterized roles in Cd tolerance and metal immobilization (Rajkumar et al., 2009; Sun et al., 2022; Kang et al., 2024). Despite this apparent structural simplification, functional prediction indicated that key processes such as carbon turnover and nitrogen fixation remained largely maintained, demonstrating strong functional redundancy embedded within the rhizosphere system even when taxonomic and network structures were perturbed by Cd stress.

Collectively, these findings indicate that *P. notoginseng* operates under a coupled plant–microbe stress buffering mechanism: moderate Cd triggers beneficial feedback loops that enhance physiological performance and microbial facilitation, whereas excessive Cd breaks network connectivity, reduces cooperative interactions, and leads to functional decline. The preferential enrichment of *Pseudomonas* and *Bacillus* suggests that these taxa may play contributory roles in plant stress mitigation under Cd exposure and highlights their relevance as candidate PGPR for future investigation and experimental validation in Cd-affected soils (Kumar et al., 2023; Ding et al., 2025).

## 5 Conclusion

Overall, these findings reveal a threshold-dependent, coupled plant–microbe buffering mechanism in *P. notoginseng*: moderate Cd (∼30 mg kg^–1^) promotes coordinated resilience between plant physiology and rhizosphere function, whereas excessive Cd disrupts cooperative microbial interactions and impairs regulatory and interaction-level processes.

The consistent enrichment of Cd-tolerant, plant-associated taxa such as *Pseudomonas* and *Bacillus* under high Cd stress suggests that these genera may play contributory roles in plant stress mitigation within the rhizosphere. However, it should be noted that these implications are based on community-level patterns and predictive analyses. Targeted isolation, functional characterization, and field validation will be required to substantiate their suitability for microbial inoculant development or phytoremediation applications in Cd-contaminated soils.

## Data Availability

The datasets presented in this study can be found in online repositories. The names of the repository/repositories and accession number(s) can be found at: https://www.ncbi.nlm.nih.gov/, PRJNA1227732.

## Footnotes

1
http://huttenhower.sph.harvard.edu/galaxy/
