# Supplementary material for: Responses of Panax notoginseng (Burk.) F.H. Chen to cadmium stress: hormetic effects on growth, antioxidant systems, and rhizosphere microbial dynamics
Source: Front Microbiol. 2026 Feb 18;17:1741415. doi: 10.3389/fmicb.2026.1741415 (PMC12957275; doi:10.3389/fmicb.2026.1741415)
Supplement: Supplementary file 1 [file Data_Sheet_1.docx]

Supplementary Material

# Supplementary Figures


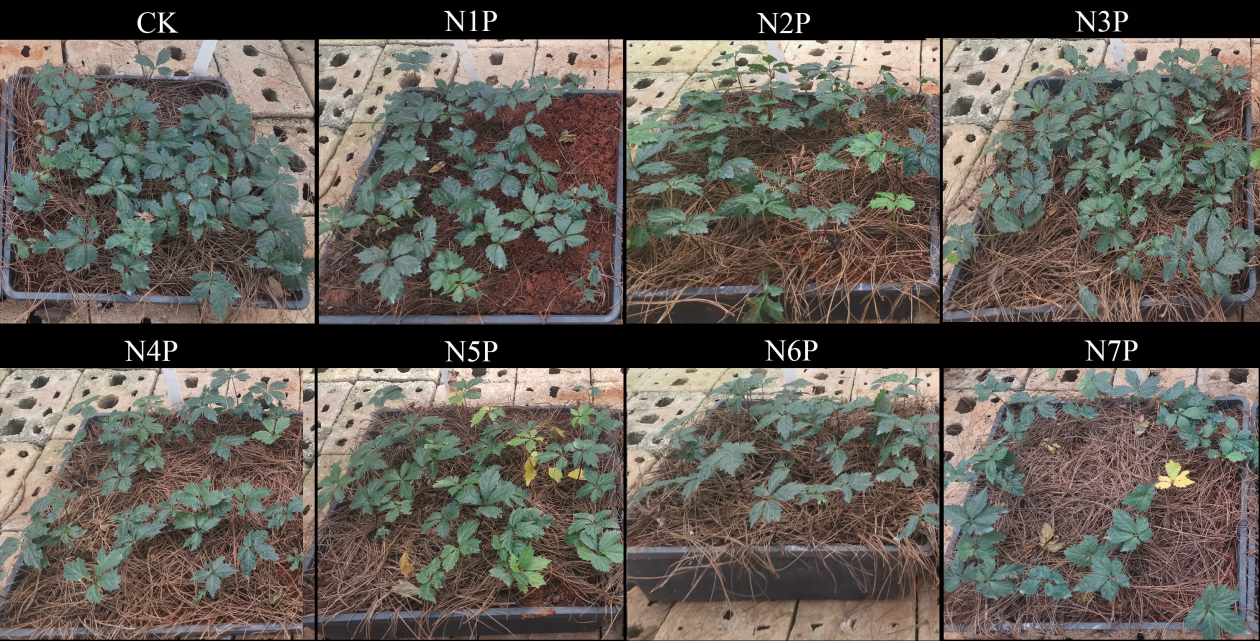


**Supplementary Figure 1.** *P. notoginseng* after 75 Days of Cadmium Stress Treatment.


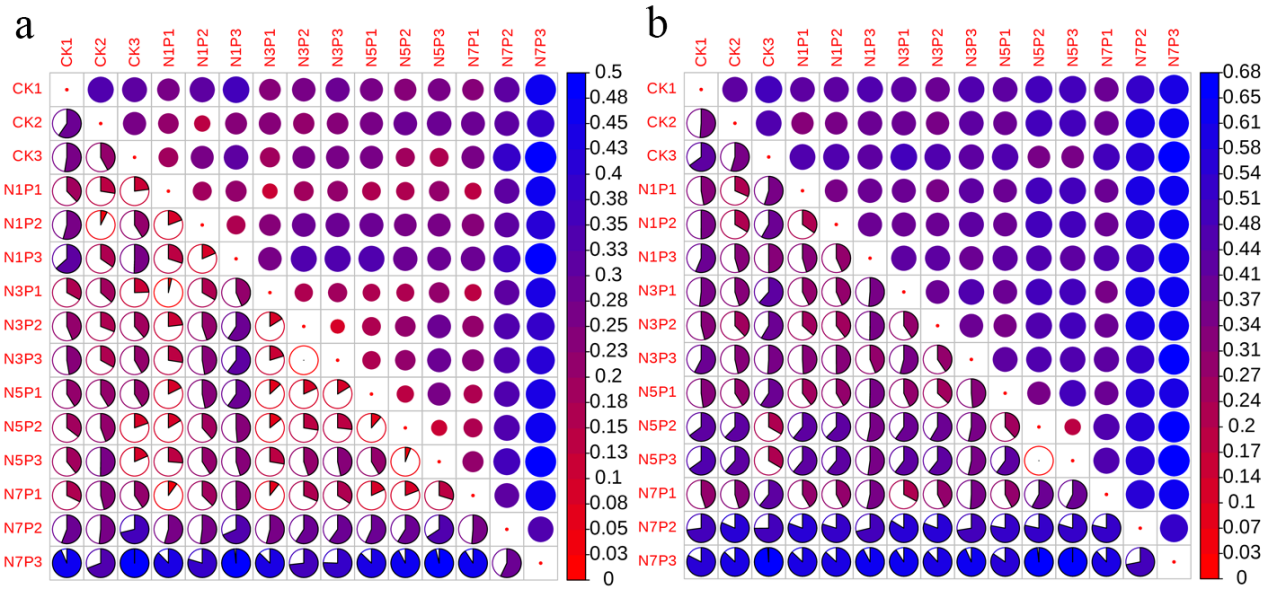


**Supplementary Figure 2.** Community dissimilarity analysis using UniFrac distances. Hierarchical clustering heatmaps based on (a) weighted and (b) unweighted UniFrac distances showing pairwise community dissimilarities. Color intensity represents distance magnitude (scale: 0-0.5). Sample clustering reveals treatment-induced changes in community structure with N7P3 showing maximum divergence from controls.


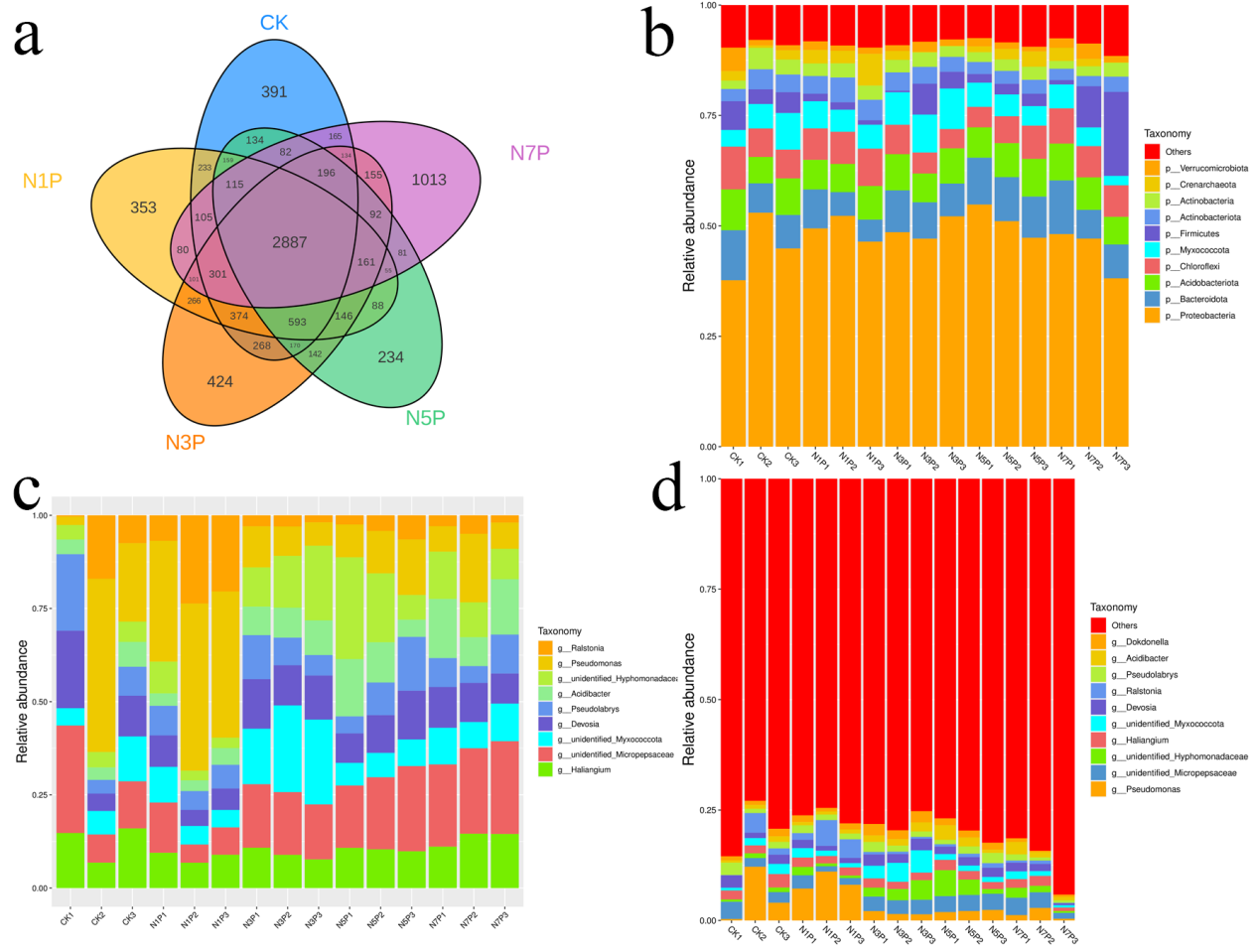


**Supplementary Figure 3.** OTU-based Wayne plots (a); plots of compositional taxonomic relative abundance of soil bacteria at the phylum (b) and genus levels (c) for the five treatment groups; plots of genus-level abundance distributions with Others removed (d).


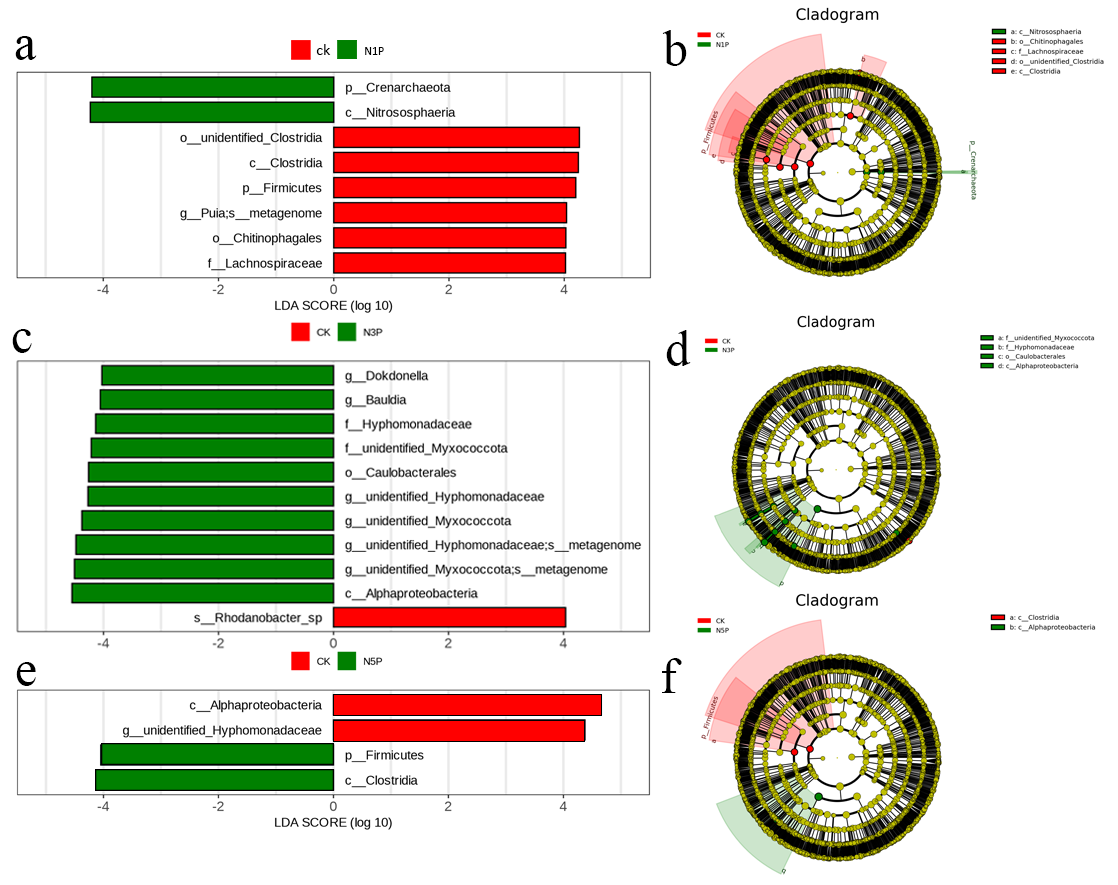


**Supplementary Figure 4.** Effects of Cd stress on soil microbial community composition. (a, c, e) LEfSe bar plots showing significantly enriched microbial taxa in different Cd treatments. (a: N1P, c: N3P, e: N5P). (b, d, f) OTU-based phylogenetic cladograms visualizing microbial community shifts under different Cd concentrations. (b: N1P, d: N3P, f: N5P).


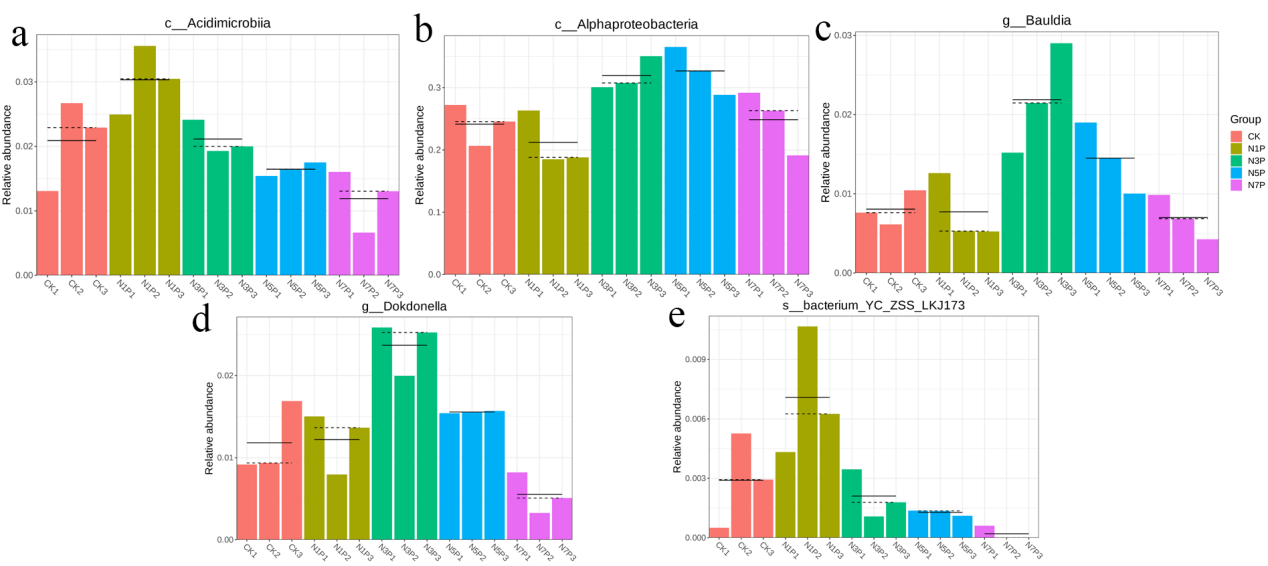


**Supplementary Figure 5.** Relative abundance of key microbial taxa under different Cd treatments. The bar plots represent the relative abundance of (a) *Acidimicrobiia*, (b) *Alphaproteobacteria*, (c) *Bauldia*, (d) *Dokdonella*, and (e) *bacterium YC ZSS LKJ173* across different Cd concentrations.


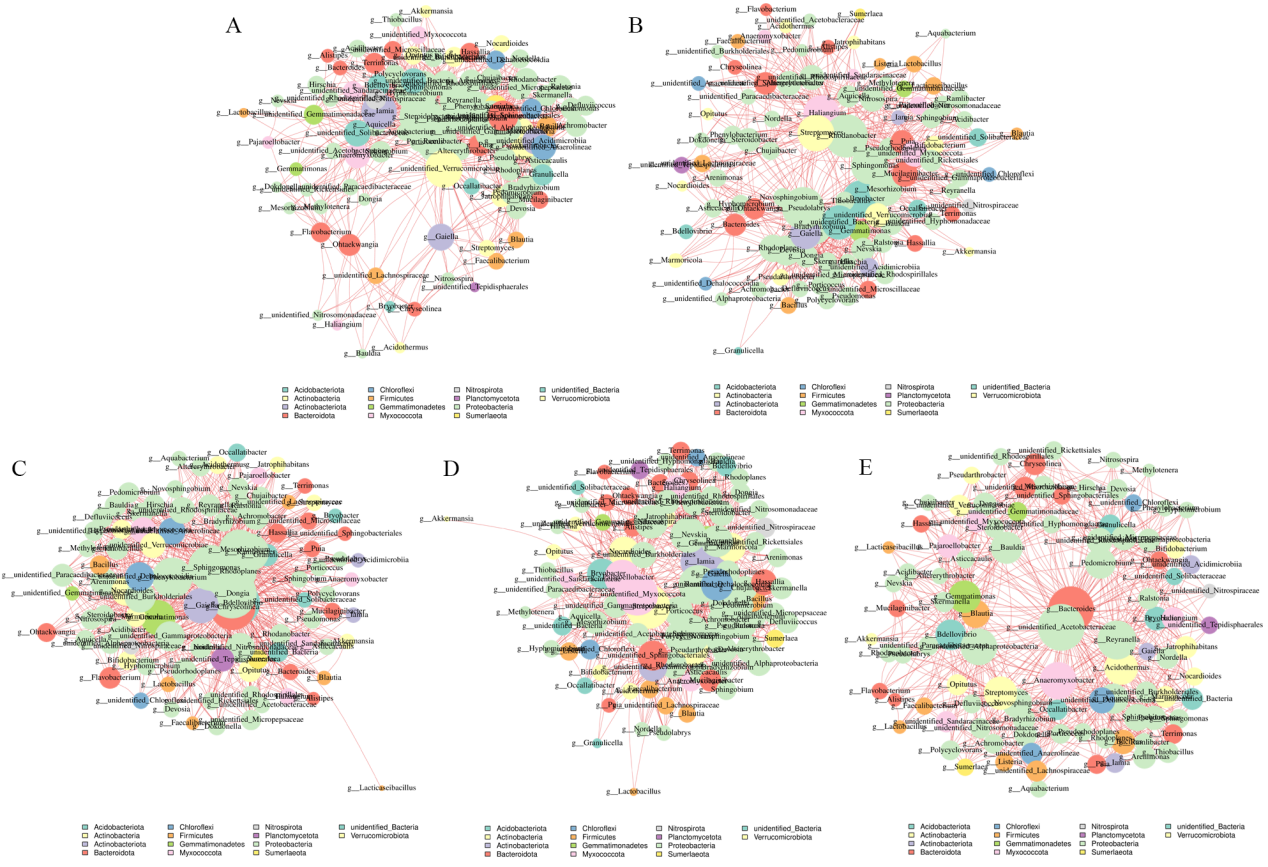


**Supplementary Figure 6.** Gradient co-expression networks under increasing Cd concentrations (A: CK, B: N1P, C: N3P, D: N5P, E: N7P).


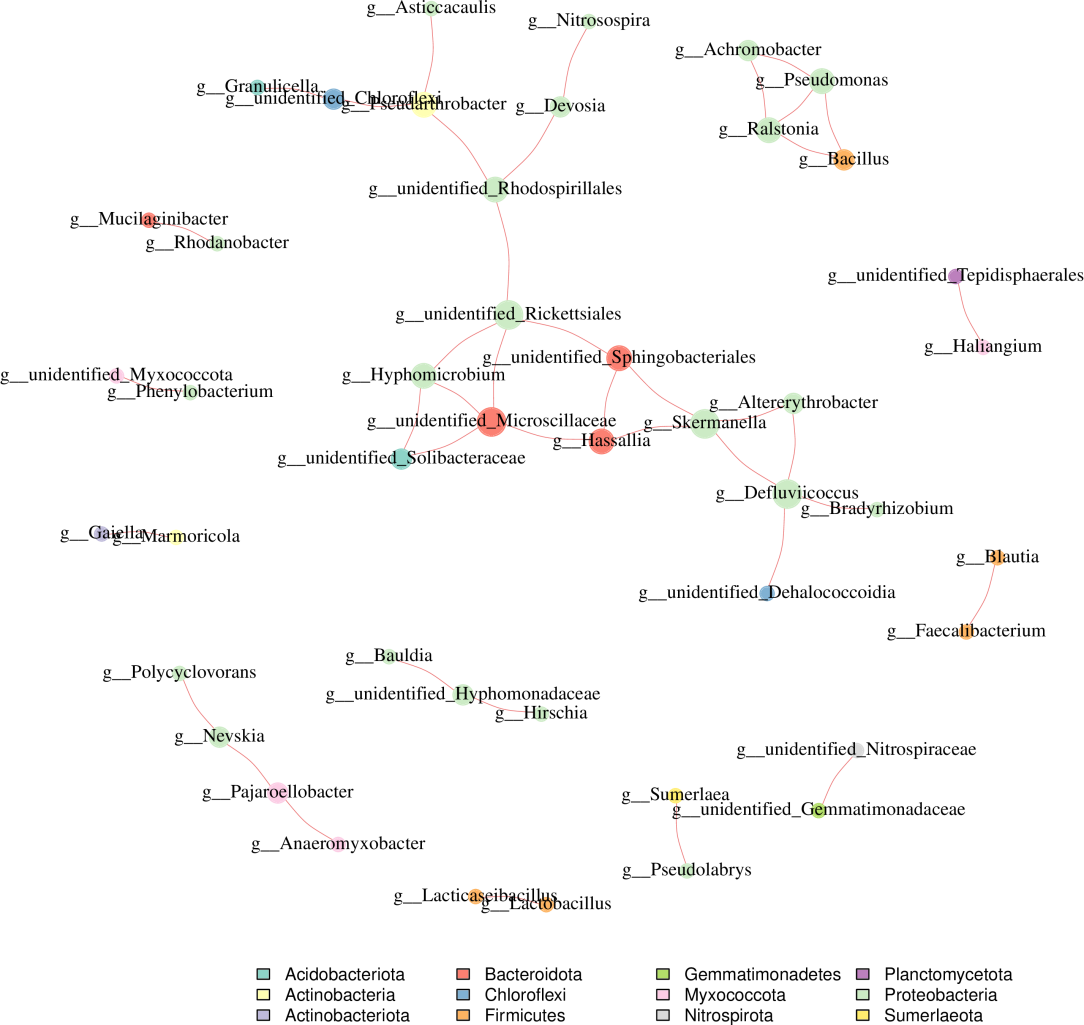


**Supplementary Figure 7.** Global co-expression network combining all samples.

**Supplementary Table 1.** Pairwise PERMANOVA (adonis) results based on UniFrac distances. Values are reported as weighted UniFrac (outside parentheses) and unweighted UniFrac (in parentheses). Df indicates model and residual degrees of freedom [1 (4)]. R^2^ represents the proportion of variance explained by treatment. Pr(>F) was obtained from permutation tests.

| **Group** | **Df** | **Sums Of Sqs** | **Mean Sqs** | **F.Model** | **R^2^** | **Pr(>F)** |
| --- | --- | --- | --- | --- | --- | --- |
| CK vs N1P | 1(4) | 0.081(0.297) | 0.081(0.07425) | 1.097 | 0.215(0.785) | 0.4 |
| CK vs N3P | 1(4) | 0.136(0.312) | 0.136(0.078) | 1.746 | 0.304(0.696) | 0.2 |
| CK vs N5P | 1(4) | 0.108(0.285) | 0.108(0.07125) | 1.509 | 0.274(0.726) | 0.3 |
| CK vs N7P | 1(4) | 0.245(0.612) | 0.245(0.153) | 1.599 | 0.286(0.714) | 0.1 |
| N1P vs N3P | 1(4) | 0.2(0.192) | 0.2(0.048) | 4.168 | 0.51(0.49) | 0.1 |
| N1P vs N5P | 1(4) | 0.165(0.165) | 0.165(0.04125) | 3.981 | 0.499(0.501) | 0.1 |
| N1P vs N7P | 1(4) | 0.315(0.492) | 0.315(0.123) | 2.557 | 0.39(0.61) | 0.1 |
| N3P vs N5P | 1(4) | 0.116(0.18) | 0.116(0.045) | 2.565 | 0.391(0.609) | 0.1 |
| N3P vs N7P | 1(4) | 0.279(0.508) | 0.279(0.127) | 2.196 | 0.354(0.646) | 0.1 |

**Supplementary Table 2.** Topological properties of genus-level co-occurrence networks under control (CK) and high Cd stress (N7P).

| **Treatment** | **Threshold** | **Total edges** | **Positive edges (%)** | **Negative edges (%)** |
| --- | --- | --- | --- | --- |
| CK | \|ρ\| ≥ 0.8, *p* < 0.05 | 792 | 501 (63.3%) | 291 (36.7%) |
| N7P | \|ρ\| ≥ 0.8, *p* < 0.05 | 862 | 455 (52.8%) | 407 (47.2%) |

Edge numbers and the proportions of positive (*ρ* > 0) and negative (*ρ* < 0) correlations were calculated from the filtered SparCC correlation tables.

**Supplementary Table 3.** Quantitative comparison of selected predicted functions across Cd treatments (mean ± SD, n=3) and one-way ANOVA.

| Function | CK | N1P | N3P | N5P | N7P | ANOVA (P) | Direction |
| --- | --- | --- | --- | --- | --- | --- | --- |
| Carbohydrate metabolism | 0.083±0.002 | 0.081±0.001 | 0.083±0.000 | 0.083±0.001 | 0.085±0.001 | >0.05 | Stable |
| Energy metabolism | 0.042±0.002 | 0.042±0.001 | 0.042±0.000 | 0.042±0.000 | 0.043±0.001 | >0.05 | Stable |
| Signal transduction | 0.026±0.002 | 0.027±0.001 | 0.026±0.000 | 0.025±0.000 | 0.026±0.001 | <0.05 | ↓ |
| Chemoheterotrophy | 0.340±0.030 | 0.356±0.009 | 0.332±0.018 | 0.316±0.005 | 0.299±0.054 | >0.05 | Stable |
| Nitrogen fixation | 0.013±0.004 | 0.013±0.001 | 0.019±0.006 | 0.018±0.001 | 0.030±0.016 | <0.05 | ↑ |
